# Comparative Review of SARS-CoV-2, SARS-CoV, MERS-CoV, and Influenza A Respiratory Viruses

**DOI:** 10.3389/fimmu.2020.552909

**Published:** 2020-09-11

**Authors:** Zeinab Abdelrahman, Mengyuan Li, Xiaosheng Wang

**Affiliations:** ^1^Biomedical Informatics Research Lab, School of Basic Medicine and Clinical Pharmacy, China Pharmaceutical University, Nanjing, China; ^2^Cancer Genomics Research Center, School of Basic Medicine and Clinical Pharmacy, China Pharmaceutical University, Nanjing, China

**Keywords:** SARS-CoV-2, SARS-CoV, MERS-CoV, influenza A virus, COVID-19

## Abstract

The 2019 novel coronavirus (SARS-CoV-2) pandemic has caused a global health emergency. The outbreak of this virus has raised a number of questions: What is SARS-CoV-2? How transmissible is SARS-CoV-2? How severely affected are patients infected with SARS-CoV-2? What are the risk factors for viral infection? What are the differences between this novel coronavirus and other coronaviruses? To answer these questions, we performed a comparative study of four pathogenic viruses that primarily attack the respiratory system and may cause death, namely, SARS-CoV-2, severe acute respiratory syndrome (SARS-CoV), Middle East respiratory syndrome (MERS-CoV), and influenza A viruses (H1N1 and H3N2 strains). This comparative study provides a critical evaluation of the origin, genomic features, transmission, and pathogenicity of these viruses. Because the coronavirus disease 2019 (COVID-19) pandemic caused by SARS-CoV-2 is ongoing, this evaluation may inform public health administrators and medical experts to aid in curbing the pandemic's progression.

## Introduction

The 2019 novel coronavirus (SARS-CoV-2), severe acute respiratory syndrome coronavirus (SARS-CoV), Middle East respiratory syndrome coronavirus (MERS-CoV), and influenza A viruses are major pathogens that primarily target the human respiratory system. Diseases associated with their infections vary from mild respiratory illness to acute pneumonia and even respiratory failure. Since 1918, the influenza A viruses have caused four pandemics. The first and most severe pandemic in recent history, known as “Spanish influenza,” occurred in 1918 and was caused by an H1N1 influenza A virus (IAV) strain (1). Approximately 500 million people were infected, and 50 million people died during this pandemic. The second pandemic, known as “Asian influenza,” occurred in 1957, was caused by an H2N2 IAV strain, and resulted in ~1.1 million deaths worldwide (2). The third pandemic, known as “Hong Kong flu,” occurred in 1968 and was caused by an H3N2 IAV strain, resulting in ~1 million deaths worldwide (3). The fourth pandemic was caused by the influenza A (H1N1) pdm09 virus, also known as the “novel influenza A virus,” and resulted in 151,700–575,400 deaths worldwide from 2009 to 2010 (4, 5). Since that time, the novel influenza A virus has continued to spread as a seasonal flu virus. From September 2019 to February 2020, this virus caused at least 34 million flu illnesses and 20,000 deaths. In November 2002, before the fourth influenza A pandemic, an epidemic caused by a betacoronavirus (SARS-CoV) and known as severe acute respiratory syndrome (SARS) began in South China and spread to 29 countries. The SARS outbreak caused ~8,000 infections and 774 deaths before it was contained in July 2003, with a case fatality rate (CFR) of 9.6% (the CFR was ~50% among patients 65 or older) (6). However, since 2004, there have not been any SARS cases reported anywhere in the world. In September 2012, Saudi Arabia reported the first case of Middle East respiratory syndrome (MERS), which was caused by another type of betacoronavirus (MERS-CoV). MERS-CoV spread to 27 countries and caused 2,519 infections and 866 deaths by January 2020, with a CFR of 34.4% (7).

**Figure 1:**
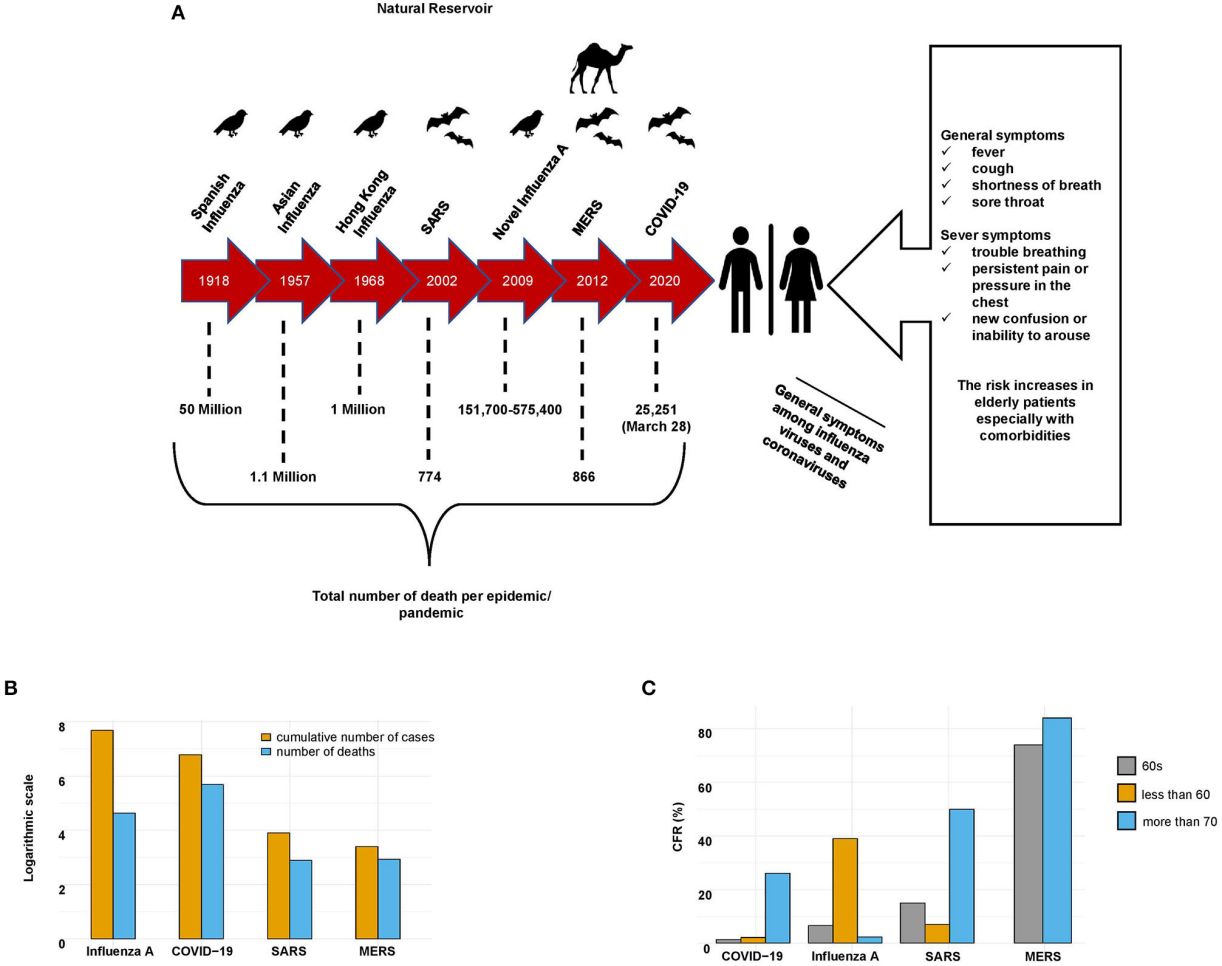
General characteristics of SARS-CoV-2, SARS-CoV, MERS-CoV, and influenza A viruses. **(A)** Epidemics of SARS-CoV-2, SARS-CoV, MERS-CoV, and influenza A viruses. The timeline, natural reservoirs, total number of deaths, and symptoms of the patients infected with these viruses. **(B)** Cumulative numbers of cases and deaths caused by SARS-CoV-2, SARS-CoV, MERS-CoV, and influenza A (during the last seasonal flu 2019–2020) viruses. Influenza A virus infected the most people, while SARS-CoV-2 caused the most deaths. **(C)** Case-fatality rate (CFR) of patients infected with SARS-CoV-2, SARS-CoV, MERS-CoV, and influenza A (the last seasonal flu 2019–2020) viruses stratified by age.

In December 2019, cases of the new coronavirus disease 2019 (COVID-19), caused by a new betacoronavirus (SARS-CoV-2), were first reported in Wuhan, China (8). These cases were characterized by acute pneumonia-associated symptoms, such as fever, dry cough, chills, shortness of breath, and muscle pain (9). The SARS-CoV-2 outbreak rapidly spread worldwide. It has infected more than 14 million individuals and resulted in more than 500,000 deaths as of 20 July 2020. In comparison with the other two coronaviruses, SARS-CoV-2 appears to be much more contagious and infectious; it has rapidly resulted in a pandemic constituting a global health emergency (Figures 1A–C).

To better understand the current COVID-19 pandemic caused by SARS-CoV-2, we have performed a comparative study between SARS-CoV-2 and past epidemic/pandemic viral infections that primarily affect the respiratory system: the influenza A viruses (H3N2 and H1N1 strains) and the two coronaviruses SARS-CoV and MERS-CoV. We have explored the genomic characteristics, transmission, reservoirs, and pathogenesis of these four pathogens. We have also considered the preventive and control measures conducted by the World Health Organization (WHO) against the spread of these pathogens. Additionally, we have elucidated how these viruses attack the immune system and the associated host immune system response. This comparative study will aid in informing public health administrators and medical experts on how to adequately distinguish between these viruses and identify the preventive and control measures recommended by the WHO against the spread of SARS-CoV-2.

A brief comparison between the four pathogenic viruses, including their characteristics, pathogenesis, and transmission, is summarized in Table 1.

**Table 1:** General characteristics of SARS-CoV-2, SARS-CoV, MERS-CoV, and influenza A viruses.

**Characteristic**	**SARS-CoV-2**	**SARS-CoV**	**MERS-CoV**	**Influenza A**
Year of the first reported case	2019	2002	2012	1918
Country/Region of the first reported case	China	China	Middle East	United States
Natural reservoir	Unclear (possibly bats)	Chinese horseshoe bats	Camels (possibly bats)	Birds
Intermediate host	Debatable (possibly pangolins) (10)	Civet cats	Dromedary camels	Pigs
Primary modes of transmission	Droplet, aerosol, and contact	Droplet, aerosol, and contact	Droplet, aerosol, and contact	Droplet, aerosol, and contact
Incubation period	2–14 days	2–7 days	2–14 days	2 days
Reproduction number (*R*_0_)	*R*_0_ = 3.1 (coefficient of determination, *r*^2^ = 0.99)	Median: 0.58; IQR: 0.24–1.18	Mean: 0.69 (95% CI 0.50–0.92)	Median: 1.27; IQR: 1.19–1.37
Host receptor	ACE2	ACE2	DPP4	Sialic acid-containing molecules
Dominant cell entry pathway	Unclear	Clathrin- and caveolae-independent endocytic pathway (11)	Cell membrane fusion (12)	Receptor-mediated endocytosis (13)
Blood test results	Lymphopenia, thrombocytopenia, leukopenia, leucocytosis, monocytosis, and low CRP (14)	Lymphopenia, thrombocytopenia, and leukopenia (15)	Leucocytosis, monocytosis, and low CRP (16)	Lymphopenia, eosinopenia, hypoferremia, decreased levels of serum CO_2_-CP, increased levels of serum CRP and serum CH50 (17)
Case fatality rate	1–3%	~15%	34.4%	0.1%

*IQR, interquartile range; CRP, C-reactive protein; CI, confidence interval; ACE2, Angiotensin-converting Enzyme 2; DPP4, Dipeptidyl peptidase-4 inhibitor; CO2-CP, carbon dioxide; CH50, Total Complement Activity*.

## Taxonomy, Structure, and Genomic Properties of the Viruses

### Influenza A

Influenza A viruses that infect humans mainly consist of two strains (H1N1 and H3N2). Both strains are characterized as enveloped, negative-sense, single-stranded RNA viruses with a total genome size of ~13.5 kb (18, 19). The influenza A virus genome consists of eight different segments, with each segment containing a region that encodes one or two proteins with specific functions, including hemagglutinin (HA), polymerase basic protein 2 (PB2), nucleoprotein (NP), polymerase basic protein 1 (PB1), neuraminidase (NA), matrix (M), nonstructural protein (NS1), and polymerase acidic protein (PA) (20, 21).

The HA protein of influenza A viruses binds to the glycoprotein terminal sialic acid and glycolipid receptors, which contain α-2,6 and α-2,3 sialic acid groups attached to galactose. Although HA is considered to be a more crucial antigenic determinant than NA, both proteins are potentially restrictive factors for viral evolution (20, 22). In addition, there are three viral polymerase proteins, PB1, PB2, and PA, encoded on segments 1, 2, and 3, respectively; these polymerase proteins form an enzyme complex that plays a role in transcription and replication. Finally, the NP protein encoded on segment 5 is used as a model to generate additional copies (23, 24).

**Figure 2:**
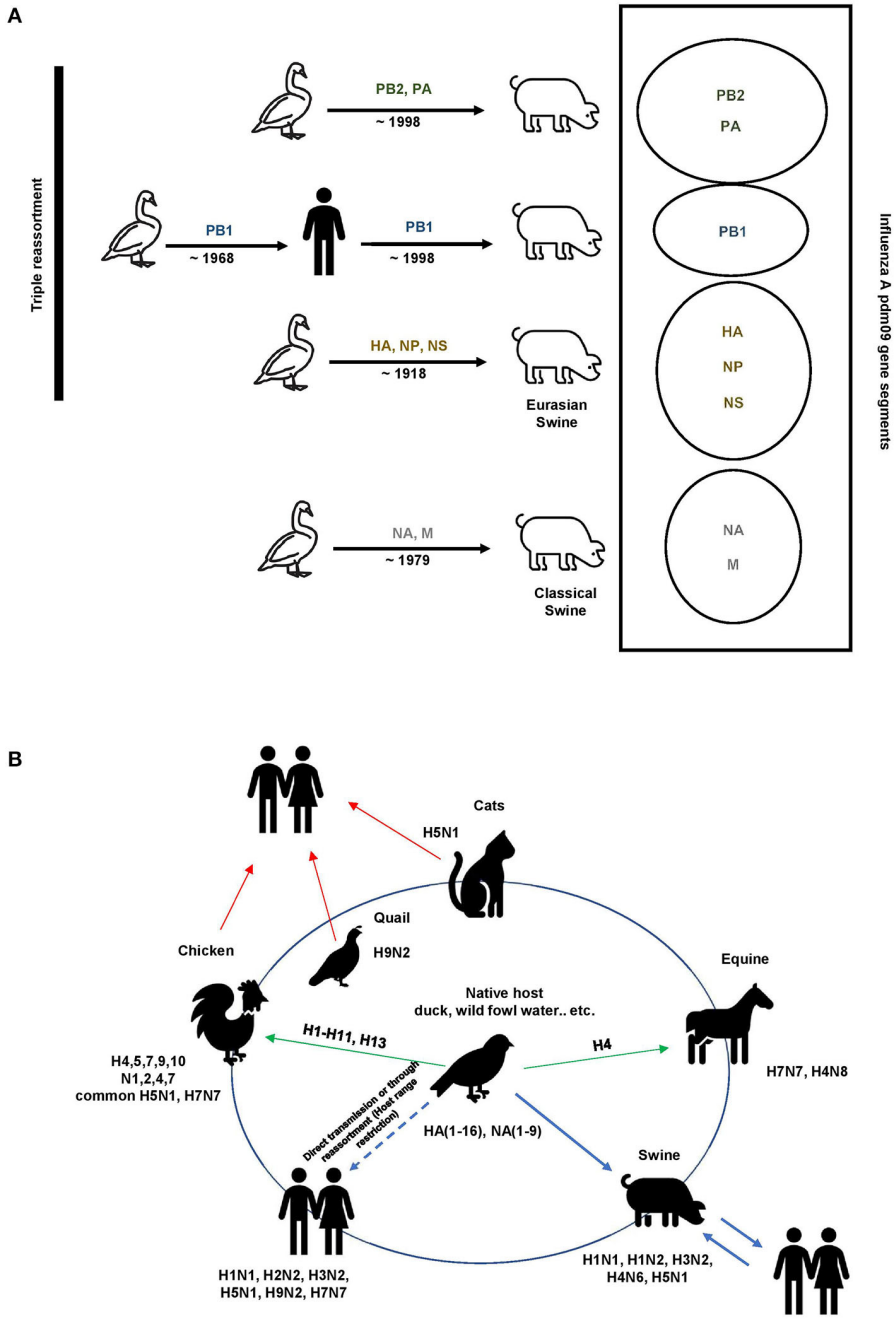
Influenza A evolution. **(A)** Triple reassortment influenza A viruses of the H1N1 subtype containing avian, swine, and human gene segments. The colored solid genes represent the gene segments as follows: yellow, classical swine A (H1N1) virus; green, North American avian virus; blue, human A (H3N2) virus; gray, Eurasian avian-like swine A(H1N1). **(B)** Reservoirs and interspecies transmission events of the pathogenic influenza A viruses. Wild birds, domestic birds, pigs, horses, and humans maintain their influenza A viruses. Spillover events occasionally occur, most frequently from wild birds (arrows in green).

Influenza A viruses exhibit antigenic drift/shift properties, allowing them to avoid the host immune response. The Centers for Disease Control and Prevention (CDC) defines antigenic drift as genetic variation that occurs in antigen structures owing to point mutations in the HA and NA genes over time, whereas antigenic shift is the result of a sudden genetic reassortment between two or more closely related influenza viral strains (23, 24). A well-known example of the antigenic shift phenomenon is the triple reassortment that occurred in the influenza A pdm09 virus and caused the 2009 pandemic as a result of the replacement of the hemagglutinin H2 and polymerase PB1 genes of the avian H2N2 virus with two new avian H3 and PB1 genes (25, 26) (Figure 2A). These antigenic drift/shift properties can potentially reduce the effectiveness of vaccines and become a considerable challenge in antiviral therapy (27, 28).

### SARS-CoV

The coronavirus family is so named because of the large spike protein molecules that are present on the virus surface and gives the virions a crown-like shape; coronavirus genomes are the largest among RNA viruses (29). This family has been classified into at least three primary genera (alpha, beta, and gamma). Within this family, seven viruses are currently known to infect humans, namely, NL63 and 229E from the alpha genus and OC43, HKU1, SARS-CoV, MERS-CoV, and SARS-CoV-2 from the beta genus. SARS-CoV is a positive-stranded RNA virus belonging to the family *Coronaviridae* (30), order *Nidovirales*, genus *Betacoronavirus*, lineage B (from the International Committee on Taxonomy of Viruses). It was characterized as a giant, enveloped, positive-stranded RNA virus with a genome comprising 29,727 nucleotides (~30 kb), 41% of which are guanine or cytosine. The genomic body of this virus has the original gene order of 5'-replicase (rep), which makes up approximately two-thirds of the genome and consists of the large genes ORF1a and ORF1b. ORF1a and ORF1b of the rep gene encode two large polyproteins known as pp1a (486 kDa) and pp1ab (790 kDa). In addition, the 3' structural spike (S), envelope (E), membrane (M), and nucleocapsid (N) proteins are encoded by four open reading frames (ORFs) downstream of the rep gene (31). The rep gene products are translated from genomic RNA, whereas the remaining viral proteins are translated from subgenomic mRNAs. In addition to the original genes, the SARS-CoV genome encodes another eight putative accessory proteins, known as ORFs 3a, 3b, 6, 7a, 7b, 8a, 8b, and 9b, which vary in length from 39 to 274 amino acids. Although the SARS-CoV rep gene and structural proteins have some sequence homology with other coronaviruses, the accessory proteins do not show substantial homology to the viral proteins of other coronaviruses at the amino acid level (31).

### MERS-CoV

Although MERS-CoV belongs to the same family, order, and genus as SARS-CoV, it was the first betacoronavirus lineage C member identified as a “novel coronavirus” with a genome size of 30,119 nucleotides. The genome of MERS-CoV encodes 10 proteins. These 10 proteins comprise two replicase polyproteins (ORF1ab and ORF1a), four structural proteins (E, N, S, and M), and four nonstructural proteins (ORFs 3, 4a, 4b, and 5) (32). In addition to the rep and structural genes, there are accessory protein genes interspersed between the structural protein genes that may interfere with the host innate immune response in infected animals (7).

### SARS-CoV-2

Although SARS-CoV-2 belongs to the same family and genus as SARS-CoV and MERS-CoV, genomic analysis revealed greater similarity between SARS-CoV-2 and SARS-CoV. Thus, researchers classified it as a member of lineage B (from the International Committee on Taxonomy of Viruses). Initially, the *Coronaviridae* Study Group of the International Committee on Taxonomy of Viruses identified this virus as a sister clade to the prototype human and bat severe acute respiratory syndrome coronaviruses (SARS-CoVs) of the species *Severe acute respiratory syndrome-related coronavirus*. Later, it was labeled as SARS-CoV-2 (33). The RNA genome size of SARS-CoV-2 is 30,000 bases in length. Among other betacoronaviruses, this virus is characterized by a unique combination of polybasic cleavage sites, a distinctive feature known to increase pathogenicity and transmissibility in other viruses (34).

Genomic analysis of SARS-CoV-2 revealed that the genome consists of six major ORFs and shares less than an 80% nucleotide sequence identity with SARS-CoV. However, the seven conserved replicase domains in the ORF1ab amino acid sequence share a 94.4% identity with those in SARS-CoV (35). Genomic analysis also revealed that the SARS-CoV-2 genome is highly similar to that of the bat coronavirus (Bat CoV RaTG13), with a sequence identity of 96.2%. Furthermore, the receptor-binding spike protein shares a 93.1% similarity to Bat CoV RaTG13 (35). Meanwhile, relative to SARS-CoV, significant differences were observed in the sequence of the S gene of SARS-CoV-2, including three short insertions in the N-terminal domain, changes in four out of five of the crucial residues in the receptor-binding motif, and the presence of an unexpected furin cleavage site at the S1/S2 boundary of the SARS-CoV-2 spike glycoprotein. This insertion is a novel feature that differentiates SARS-CoV-2 from SARS-CoV and several SARS-related coronaviruses (SARSr-CoVs) (36).

## Viral Origin and Evolution

### Influenza A

Influenza A H1N1 and H3N2 subtype viruses are two of the three combinations known to have circulated widely in humans and to currently cause seasonal influenza; these strains originated from birds and swine. Before 1979, the only lineage detected in swine herds from Europe was the classical swine influenza virus A H1N1 lineage 1A (25). This strain shares a mutual ancestor with the virus that caused the 1918 human influenza A pandemic. However, in the early 1980s, the classical swine H1N1 strain was displaced by a new European enzootic swine influenza A viral strain: the Eurasian, avian-like H1N1 (H1_av_N1) lineage 1C (26). After its rapid transmission from birds to mammals, the H1_av_N1 virus underwent rapid and sustained adaptation in mammals. Furthermore, this virus has also undergone rapid reassortment, resulting in the appearance of multiple genotypes. The two primary enzootic subtypes are H1N2 (H1huN2) lineage IB and H3N2, which occurred through the acquisition of HA or NA gene segments originating from seasonal human influenza viruses (Figure 2B) (37).

As previously mentioned, influenza A exhibits antigenic drift/shift phenomena resulting from the HA protein's ability to undergo rapid evolution because of the plasticity of the viral RNA-dependent RNA polymerase. It is believed that mutations occurring in the HA protein, including reassortments and mutations among animals and humans, were the drivers of previous pandemics (38).

Adaptive mutations can lead to a number of phenotypic changes, including variations in antigenicity, increased diversity in viral protein sequences, the ability to avoid antibody pressure, receptor preference, virulence, altered fusion functionality, and evasion of the immune response. Rapid modifications can give rise to new strains with features that are different from any viruses that have previously been confronted, potentially causing another epidemic/pandemic (38).

### SARS-CoV

In the early stages of the SARS outbreak, most of the new patient cases had animal exposure before developing the disease. Wide-ranging investigations revealed that SARS-CoV strains were transmitted to palm civets from other animals (39–41). Later, two studies reported the discovery of coronaviruses related to human SARS-CoV, which were named SARS-like coronaviruses or SARSr-CoVs, in horseshoe bats (genus *Rhinolophus*) (42, 43). Another study revealed that the viral strains of the SARS-like coronaviruses contain all of the genetic elements that are needed to form SARS-CoV. In particular, the bat strain WIV16, the closest relative to SARS-CoV, likely occurred through recombination of two other prevalent bat SARSr-CoV strains. These results suggest that bats may be the natural reservoirs for the virus and that palm civets are only intermediate hosts (Supplementary Figure 1) (44, 45).

Thus, the hypothesis formed was that the direct ancestor of SARS-CoV was produced by recombination within bats and then transmitted to palm civets or other mammals via fecal–oral transmission. When virus-infected civets were transported to Guangdong market, the virus spread among the civets in the market and underwent further mutations before transmission to humans (46).

### MERS-CoV

Unlike the SARS cases, most of the MERS cases had previous contact with dromedary camels. The MERS-CoV strains isolated from camels were almost identical to those isolated from humans (47, 48), and the MERS-CoV isolates were found to be highly prevalent in camels from the Middle East, Africa, and Asia (49, 50). Genomic sequence analysis indicated that the *Tylonycteris* bat coronaviruses HKU4 and HKU5 are phylogenetically related to MERS-CoV (they are all representatives of betacoronavirus lineage C) (51). Generally, all of the related MERS-CoVs isolated from bats support the hypothesis that MERS-CoV originated from bats (Supplementary Figure 1) (46).

### SARS-CoV-2

Before the epidemic outbreak of COVID-19 in late January 2020, several patients had been exposed to different animals (from wild animals to poultry) at the Huanan seafood wholesale market. When the CDC declared the situation to be an epidemic, several studies identified potential reservoirs, but at present, the origin and evolution of SARS-CoV-2 remain debatable. The earliest genomic sequence analysis of SARS-CoV-2 indicated that it is a member of the genus *Betacoronavirus* and falls within the subgenus *Sarbecovirus*, which also includes SARS-CoV (9, 35, 52–54). As mentioned above, preliminary comparisons revealed that SARS-CoV-2 has an almost 79% similarity with SARS-CoV at the nucleotide sequence level and a 96% similarity with horseshoe bat RaTG13 (55–57). Correspondingly, a comparative study between the RmYN02 virus from *Rhinolophus* bats in Yunan Province, China, and SARS-CoV-2 indicated that RmYN02 was the closest relative to the long replicase gene of SARS-CoV-2 (~97% nucleotide sequence similarity) (35, 36).

Even though bats are likely to be the reservoir host for this virus, their general biological differences from humans make it feasible that other mammalian species acted as intermediate hosts, in which SARS-CoV-2 obtained some or all of the mutations needed for effective human transmission. One of the suspected intermediate hosts, the Malayan pangolin, harbors coronaviruses showing high similarity to SARS-CoV-2 in the receptor-binding domain, which contains mutations believed to promote binding to the angiotensin-converting enzyme 2 (ACE2) receptor and demonstrates a 97% amino acid sequence similarity. By contrast, the genomic similarity was more divergent from SARS-CoV-2 (~91%) at the whole genome level (Supplementary Figure 1) (58, 59).

Coronaviruses have lower mutation rates than other RNA viruses, especially influenza A viruses, and high rates of viral replication within hosts because of the 3′-to-5′ exoribonuclease activity associated with the nonstructural protein nsp.14 (36, 60). This protein has an RNA proofreading function and is responsible for coronaviruses' resistance to RNA mutagens (60, 61).

## Receptor Binding of Viruses

The high unpredictability among influenza A viral strains and their HAs relates to the significant discrepancy among host cells in showing different vulnerabilities to viral infection. HA plays a role in mediating the binding of influenza A viruses to sialic acid host cell receptors (62). The receptor-binding site lies at the top of the R domain of HA and contains exceptionally variable antigenic binding loops (63). Once the virus is bound to the host receptor, endocytosis of the virus element occurs. Additionally, a pH-dependent membrane fusion process is significant in controlling the viral genome's release into the host cell. Influenza A viral strains and their HAs are very variable, which contributes to the significantly different vulnerabilities of host cells to viral infection (64).

Influenza A viruses have demonstrated dominant genomic mutations, such as those within the HA 220 loop (Q223) and the D222G and D222N mutations, in which aspartic acid (D) is replaced by glycine (G) or asparagine (N), respectively. The D222G mutation is responsible for a change in receptor-binding affinity that enables the virus to bind to α-2,6 and α-2,3 sialic acid receptors on the epithelial cells of the upper respiratory tract and ciliated epithelial cells in the lower respiratory tract, respectively (65, 66).

Although HA plays a crucial role in receptor binding and concurrent mutation capabilities, NA also has a key role in removing sialic acids from cellular receptors and from the new HA and NA on budding virions, which are sialylated as part of the glycosylation processes within the host cell (67). A balance between HA and NA is essential for viral fitness. Any mutations in HA or environmental changes, such as low pH conditions, can affect NA's activity against sialoglycans (68, 69).

The SARS-CoV trimeric spike protein facilitates coronavirus entry into host cells by binding to the host receptor and subsequently fusing the viral and host membranes. The spike protein consists of three segments, one of which is the ectodomain (70). The ectodomain is composed of two subunits: S1 and S2. The S1 subunit contains two individual domains, an N-terminal domain (NTD) and a C-domain, and each NTD or C-domain (sometimes both) binds to the host receptor to function as the receptor-binding domain (RBD). ACE2 is the host cell receptor of SARS-CoV and the primary target of deactivating antibodies. Several studies have shown that the binding affinity between the RBD of each SARS-CoV strain and ACE2 positively correlates with the contagion of different SARS-CoV strains in host cells (Supplementary Figure 2) (71, 72).

The MERS-CoV spike protein subunit S1 C-domain has also been identified as the RBD (73). However, unlike SARS-CoV, MERS-CoV uses a dipeptidyl peptidase 4 (DPP4) β-propeller as its receptor. Likewise, the RBD of MERS-CoV contains an accessory subdomain that functions as the receptor-binding motif (RBM). Although the RBD core structures are remarkably analogous between MERS-CoV and SARS-CoV, their RBMs are distinct and may result in the recognition of different receptors (Supplementary Figure 2) (73).

Since the outbreak of SARS-CoV-2, several studies have analyzed its genome and compared it with other coronaviruses, such as MERS-CoV and SARS-CoV (74, 75). The results of these studies have shown that SARS-CoV-2 has a similar RBD structure to that of SARS-CoV, despite amino acid variations at some key residues (9). Genomic comparison of SARS-CoV-2 with SARS-CoV and bat SARS-like coronaviruses revealed that the S1 subunits of the spike proteins have a sequence identity of ~75%, and recent experimental studies confirmed that ACE2 is the human receptor of SARS-CoV-2 (34). Therefore, it is essential to characterize the human receptor-binding capacity of SARS-CoV-2 to evaluate its human–human transmissibility. A recent study used the protein–protein docking method to measure the interaction between the SARS-CoV-2 spike RBD and ACE2; it was revealed that the SARS-CoV-2 human receptor-binding affinity was 73% of that of SARS-CoV, which suggests that SARS-CoV-2 binds to ACE2 with intermediate affinity (76) (Supplementary Figure 2).

## Host Factors, Disease Severity, and Pathogenesis

Influenza, SARS, and MERS have caused major global health threats, and now the COVID-19 pandemic is rapidly spreading worldwide and is having a widespread and profound impact. Both viral and host factors determine the severity and clinical outcomes of the diseases caused by these viruses. Host factors include host immunity, age, sex, morbidities, and genetic variations.

Influenza infections can cause high morbidity and mortality rates in the elderly (65 or older) and young populations with comorbidities (Figure 1C). Pathogenesis following influenza A infection occurs in two stages. The first stage is defined by the peak viral titer, along with the peak amount of inflammation associated with the infection, and lasts ~1 to 3 days. In the second stage, the infection progresses in some patients, and in severe cases, it may be associated with acute respiratory distress syndrome and sometimes death (77). Once a patient is infected with an influenza A virus, the humoral immune response will release neutralizing antibodies to target the influenza HA protein by blocking the binding of HA to sialic acids, thereby preventing viral fusion, inhibiting the release of offspring virions, and delaying proteolytic cleavage of HA by host receptors (78).

Once a patient is infected with SARS-CoV, MERS-CoV, or SARS-CoV-2, the host innate immune system will identify the virus by using pattern recognition receptors, such as a toll-like receptor, NOD-like receptor, or RIG-I-like receptor, to recognize pathogen-associated molecular patterns. The adaptive immune response also plays a significant antiviral role by stabilizing the host defense mechanism against pathogens and minimizing the risk of developing an autoimmune reflex response or inflammation (9, 79). In general, human coronaviruses can be classified into two types: lowly pathogenic and highly pathogenic. Viruses with low pathogenicity, including HCoV-229E, HCoV-OC43, HCoV-NL63, and HCoV-HKU, can cause mild upper respiratory tract infections. In contrast, highly pathogenic viruses, including SARS-CoV, MERS-CoV, and SARS-CoV-2, can cause lower respiratory tract infections, severe pneumonia, and sometimes fatal acute lung injury or acute respiratory distress syndrome, especially in older individuals (≥65 years old) (Figure 1C) (80).

In addition to the lungs, coronavirus infection may damage other organs or tissues, including the gastrointestinal tract (81), spleen, lymph nodes, brain, skeletal muscles, thyroid, and heart (82, 83). The destruction of lung cells prompts a local immune response, engaging macrophages and monocytes that respond to the infection, release cytokines, and enhance adaptive T and B cell immune responses. In some cases, a dysfunctional immune response occurs, which can cause severe lung and systemic pathology. The invading coronavirus may incite host immune responses, and an excessive immune response may cause immunopathological damage (known as a cytokine storm) in patients with coronavirus infections (9, 84). Cytokine storms may enhance the infiltration of non-neutralizing antiviral proteins that facilitate viral entry into host cells, leading to increased viral infectivity (82, 85). Therefore, cytokine storms play a key role in the pathogenesis and clinical outcomes of patients with coronavirus infection.

## Transmissibility and Virulence

The initiation of a pandemic requires the rise of a virus in a human population in which there is little or no pre-existing immunity, and the virus must be able to persist through human-to-human transmission (86, 87). The ability of influenza A viruses to adapt to various hosts and undergo reassortment events ensures the constant generation of new strains. These strains have variable degrees of pathogenicity, pandemic transmissibility, and reproduction numbers (*R*_0_) (Table 1) (88). However, only three subtypes of influenza A (H1–H3) have acquired the properties to cause pandemics in the last two centuries. Thus, an understanding of the capability of a virus to attain a contagious phenotype is a critical factor in evaluating the pandemic potential of novel subtypes (89, 90). The use of animal models has facilitated detailed studies of influenza A virus transmission by the contact and respiratory droplet routes. The presence of a single sick individual in a small space, such as an airplane or room, has been shown to be adequate for an outbreak among healthy individuals (Supplementary Figure 3) (91). Although infection and case fatality rates vary from one pandemic to another, the rates of influenza A virus infections in the pandemics were high, especially among people with little to no pre-existing immunity. When pandemic viruses become established in humans, their effective seasonal spread among healthy individuals eventually provides an enduring and even more significant public health issue in terms of hospitalizations and, in some cases, fatalities. Particle size (92), the distance of spread (92), disposition (92, 93), temperature (94), and relative humidity (95) are all considered to be factors that influence the rate of transmissibility of influenza A viruses. In addition, sialic acid receptors (α-2,3 and α-2,6) can affect the general species-specific cellular tropism of influenza A viruses (63).

Contaminated surfaces also play an essential role in transmission. A respiratory pathogen can survive on surfaces, be transferred to hands or other equipment, and initiate infection through contact with the eyes, nose, or mouth (Supplementary Figure 3) (96). Influenza A has been shown to survive for 24–48 h on stainless steel and plastic surfaces. Inversely, the strains survived for <8–12 h on cloth, paper, and tissues. Quantifiable amounts of influenza A viruses were observed to be transmitted from stainless steel surfaces to hands after 24 h and from tissues to hands for up to 15 min. Viruses also survive on hands for up to 5 min after transfer from environmental surfaces. These results indicate a high transmission rate for influenza A viruses (97).

SARS-CoV, MERS-CoV, and SARS-CoV-2 can survive on surfaces for extended periods, sometimes up to months. Like the influenza A viruses, the factors affecting the survival of these viruses on surfaces include the strain variation, titer, surface type, mode of deposition, temperature, humidity, and method used to determine the viability of the virus (98, 99). Several studies have indicated that SARS-CoV, MERS-CoV, and SARS-CoV-2 can survive on dry surfaces for a sufficient duration to accelerate onward transmission. Viable MERS-CoV was detected on steel and plastic surfaces after 48 h at 20°C with 40% relative humidity, with a decreased viability of about 8 h at 30°C with 80% relative humidity and of about 24 h at 30°C with 30% relative humidity. The estimated half-life of MERS-CoV ranges from ~0.5 to 1 h (98). On the other hand, another study conducted on the viability of SARS-CoVs detected on plastic surfaces and on polystyrene Petri dishes revealed that the virus survived for more than 5 days and more than 20 days, respectively, at room temperature. The viral viability was constant at lower temperatures (28°C) and lower humidity (80–89%) (100), whereas survival times ranged from 5 min to 2 days on paper, disposable gowns, and cotton gowns (99).

Since the SARS-CoV-2 outbreak began, several researchers have attempted to analyze the survival time of this virus on different surfaces. One study published in the middle of March 2020 analyzed the aerosol and surface stabilities of SARS-CoV-2 and SARS-CoV. The study utilized five different environments (aerosols, plastic, stainless steel, copper, and cardboard). The results showed that the half-lives of SARS-CoV-2 and SARS-CoV were similar in aerosols and on copper. However, on cardboard surfaces, the half-life of SARS-CoV-2 was longer than that of SARS-CoV, and the highest levels of viability for both viruses were observed on stainless steel and plastic (~5.6 h on stainless steel and 6.8 h on plastic). The researchers concluded that the differences in the epidemiological characteristics of these viruses could result from other factors and that aerosol and fomite transmission of SARS-CoV-2 is probable because the virus can remain viable and infectious in aerosols and on surfaces for hours and hours to days, respectively (101).

The effective management and control of such infections are increasingly performed with extensive contributions from mathematical modeling, which not only provides information on the nature of the infection itself but also makes predictions about the likely outcome of alternative courses of action (102). One useful mathematical model is the reproductive number *R*_0_, which is defined as the average number of secondary cases generated per typical infectious case (103). A value of *R*_0_ > 1 indicates that the infection may persist or grow in the population, whereas a value of *R*_0_ < 1 indicates that this infection will decrease in the population, although exceptions occur (103). The majority of seasonal influenza *R*_0_ values have been calculated for different populations and different continents, such as Europe and North America, with a median point estimate of *R*_0_ = 1.27 (IQR: 1.19–1.37) (104). The initial estimations of the reproduction numbers of SARS-CoV and MERS-CoV were calculated for China and the Middle East with *R*_0_ median = 0.58 (IQR: 0.24–1.18) (105) and *R*_0_ mean = 0.69 (95% CI: 0.50–0.92) (106), respectively. However, among the four viruses, SARS-CoV-2 has been calculated to be the most contagious, such as the *R*_0_ value associated with the Italian outbreak with a median point estimate of *R*_0_ = 3.1 (coefficient of determination, *r*^2^ = 0.99) (107).

## Prevention, Control, and Treatment of Virus Infection

Strategies for preventing and controlling pandemic/epidemic viruses can be improved by being well-prepared. Preparedness strategies, which primarily include the quarantine of infected persons, self-protection (wearing facemasks, using disinfectants, washing hands, and disinfecting surfaces with bleach or alcohols), and social distancing are all considered to be important for a comprehensive plan that can be tested and promoted by conducting exercises to engage the whole of society.

An influenza pandemic can be catastrophic, and in a typical year of seasonal outbreaks, influenza A viruses cause as many as 5 million cases of severe illness in humans and over 500,000 deaths. After the first confirmed cases of H1N1 influenza appeared in Mexico in February 2009, cases began to spread to the United States, and by the end of April 2009, cases had been reported in several United States cities and other countries on various continents, such as Canada, the United Kingdom, and New Zealand (108). During the last pandemic, the first activation of the International Health Regulations (IHR) provisions was prompted. The discussions that led to the IHR implementation were based on the SARS outbreak experience in 2003. These regulations describe the responsibilities of individual countries and the leadership role of the WHO in declaring and managing a public health emergency of international concern, establishing systematic approaches to surveillance, promoting technical cooperation, and sharing logistic support (108). However, because of the significant diversity of influenza viruses in animal hosts, extensive experimental testing and the development of pandemic preparedness measures against all viruses is unachievable (109).

In this regard, the WHO periodically updates the influenza risk management and preparedness plan, and the latest guidance document, *Pandemic Influenza Risk Management* (PIRM), was released in May 2017 (110). This updated document supports national and global pandemic preparedness and risk management and utilizes lessons learned at the country, regional, and global levels (110). Furthermore, several WHO preparedness documents have been released since PIRM, such as *Essential steps for developing or updating a national pandemic influenza preparedness plan* (released in March 2018) and *A practical guide for developing and conducting simulation exercises to test and validate pandemic influenza preparedness plans* (published in September 2018) (111).

During the SARS epidemic, more than 8,000 people were infected, and 774 deaths occurred between November 2002 and December 2003. SARS is highly contagious and is transmitted primarily by respiratory droplets; the highest transmission rates of SARS occurred in healthcare facilities (112). At the end of the SARS outbreak, the cases of over 1,700 healthcare workers who had been affected were reported to the WHO, from China (19% of total cases), Canada (43%), France (29%), and Hong Kong (22%). During this epidemic, insufficient or inappropriate infection control measures, such as inconsistent use of personal protective equipment, reuse of N95 masks, and lack of adequate infection control, were related to the high risk of infection among healthcare workers (113). Thus, in 2004, after the epidemic was contained, the WHO released a framework that was prepared according to the six phases of an epidemic, moving from preparedness, planning, and routine surveillance for cases, through to the prevention of the consequent international spread, to the disruption of global transmission (114).

Since 2012, 27 countries have reported cases of MERS; Saudi Arabia has reported ~80% of human cases, and more than 50% of the cases in healthcare workers were nurses (115). The WHO, in collaboration with the Food and Agriculture Organization of the United Nations (FAO), the World Organization for Animal Health (OIE), and national governments, have been working with healthcare workers and scientists in affected countries to gather and share scientific evidence based on the previous coronavirus epidemic. This information gathering process has been beneficial for better understanding of the virus and the disease it causes and for the regulation of outbreak response priorities, treatment approaches, and clinical management tactics (113).

Although accumulated knowledge and risk preparedness from the influenza pandemics and SARS/MERS epidemics allowed researchers to examine the effectiveness of strategic plans in dealing with the ongoing pandemic of COVID-19, several challenges have been raised in preventing the spread of COVID-19, such as the lack of medical supplies and laboratory facilities for the assessment of the disease and the presentation of a high number of asymptomatic cases. In response to the announcement of the emergency, governments were bound by the IHR to disclose vital information regarding the identification and detection of COVID-19, regardless of the causative agent. Within the context of the Global Humanitarian Response Plan, a Health Cluster platform has been created to assess the response to the COVID-19 pandemic worldwide. This framework has adopted the following strategies: contain the spread of the COVID-19 pandemic and decrease morbidity and mortality; decrease the deterioration of human assets and rights, social cohesion, and livelihoods; and protect, assist, and advocate for refugees, internally displaced people, migrants, and host communities who are particularly vulnerable to the pandemic (source: WHO). The primary goal of the Health Cluster is to coordinate and support partners to fulfill essential health services to achieve the framework strategies. This goal is achieved by different roles and tasks, such as by raising awareness, alertness, and response planning at the country level and by conducting training and simulation exercises. The WHO Health Cluster framework is a gateway to useful resources to support COVID-19 preparedness and response (116).

Generally, each pandemic/epidemic has presented a public health emergency of uncertain scope and effect; thus, essential elements of current approaches to pandemic preparedness and extenuation, such as the development of vaccines and stockpiling of antiviral drugs, necessitate detailed virological and immunological data on viruses with apparent pandemic potential. However, the development of vaccines against new strains is challenging. Therefore, physicians and health workers have found themselves facing the massive challenge of preventing infections or stabilizing patients' conditions. Thus, several promising attempts have been made to utilize different antiviral treatments that have already been approved by the U.S. Food and Drug Administration (FDA) for the treatment of viral pneumonia infections. A list of antiviral drugs and vaccine approaches for influenza viruses, SARS-CoV, MERS-CoV, and SARS-CoV-2 that have been used in clinics or are undergoing clinical trials are summarized in Table 2.

**Table 2:** List of antiviral drugs and vaccine approaches for SARS-CoV-2, SARS-CoV, MERS-CoV, and influenza viruses.

**Virus**	**Drug or vaccine**	**Drug mechanism of action/comments**
SARS-CoV-2	Bevacizumab, Chloroquine phosphate, Methylprednisolone, Fingolimod, Favipiravir, Lopinavir and ritonavir, Remdesivir, mRNA-1273^*^, ChAdOx1 nCoV-19^*^	**Bevacizumab:** inhibiting vascular endothelial growth factor (VEGF), which is higher in COVID-19 patients than in healthy controls; VEGF is the most potent vascular permeability inducer that induces hypoxia and severe inflammation **Chloroquine:** increasing endosomal pH, which is required for virus fusion; interfering with the glycosylation of cellular receptors of SARS-CoV; suppressing the production or release of tumor necrosis factor α and interleukin 6 **Glucocorticoids:** suppressing “cytokine storms” **Fingolimod:** preventing acute respiratory distress syndrome development **Favipiravir:** based on the results of two trials conducted in Wuhan and Shenzhen, China recommended this drug as a treatment approach for COVID-19 **Lopinavir/ritonavir:** reducing viral replications in patients infected with SARS and MERS; ritonavir reduces the first pass metabolism of lopinavir to increase its bioavailability **Remdesivir:** antiviral drug against a wide array of RNA viruses that works by combining with the nascent viral RNA chains to result in premature termination, reducing virus infections (82, 117–122)
SARS-CoV	Ribavirin, Methylprednisolone, Interferons, Lopinavir and ribavirin, Pentaglobin^*^	**Ribavirin:** preventing replication of RNA and DNA viruses **Methylprednisolone:** using interferons plus corticosteroids to reduce disease-associated impaired oxygen saturation, radiographic lung abnormalities, and creatine kinase levels (controversial arguments about using corticosteroids in SARS) **Interferons:** reducing viral replication **Lopinavir and ribavirin:** blocking the final step of virion assembly; reducing the peak viral load and the associated immunopathological damage (123)
MERS-CoV	Ribavirin and interferon-α2a, Lopinavir/ritonavir, Convalescent plasma^*^	**Ribavirin:** combining interferon-α2b to reduce MERS-CoV replication **Lopinavir/ritonavir:** improving the outcomes of MERS-CoV infection; improving pulmonary function but not reducing virus replication or severe lung pathology (124)
Influenza A virus (drugs recommended by CDC to treat flu in the 2019–2020 season)	Oseltamivir phosphate, Zanamivir, Peramivir, Baloxavir marboxil, flu vaccines (such as flu shots, nasal spray flu vaccine, quadrivalent influenza)	**Oseltamivir:** blocking neuraminidases on the surfaces of influenza viruses; interfering with host cell release of complete viral particles **Zanamivir:** inhibiting influenza A and B virus neuraminidases; preventing the release of progeny viruses from host cell surfaces; inhibiting viral replication **Peramivir:** inhibiting influenza virus neuraminidases **Baloxavir marboxil:** inhibiting polymerase acidic endonuclease, an enzyme essential for viral replication; being a prodrug converted by the hydrolysis of baloxavir **Flu vaccines:** including flu shots, nasal spray flu vaccine (FluMist Quadrivalent), quadrivalent influenza vaccine, flu vaccination by jet injector, Fluzone high-dose seasonal influenza vaccine, flu vaccine with adjuvant (FLUAD), cell-based flu vaccines (Flucelvax Quadrivalent), recombinant influenza vaccine, and intradermal influenza vaccination (125, 126).

* *Indicates that the drug is under investigation; otherwise, it has been approved by the FDA*.

## Discussion and Conclusion

Although the mode of transmission for SARS-CoV-2 is still somewhat unclear, all four viruses are thought to be transmitted by the same mechanism. Infection via respiratory droplets or secretions of infected individuals is the primary mode of transmission between humans. The spread of infection is occurring more rapidly for the current outbreak than in the SARS and MERS epidemics, although rates of human-to-human transmission were generally lower for MERS.

The CFRs across the four viruses range from 0.1 to 35% (Table 1), with the highest rate for MERS cases and the lowest for seasonal influenza; however, it is essential to note that the CFR for COVID-19 should be interpreted carefully because the outbreak is still ongoing.

With the exception of the influenza A viruses, the other viruses (SARS-CoV, MERS-CoV, and SARS-CoV-2) are similar in zoonotic transmission. The MERS-CoV reservoir hosts are dromedary camels, and the SARS-CoV reservoir hosts are likely bats. It is still unclear whether SARS-CoV-2 was zoonotically transmitted from an infected palm civet, snake, or other animal at the Chinese seafood market.

Regarding the origin of the virus, SARS-CoV and SARS-CoV-2 originate from China and share a high degree of similarity, including exposure to wild animals, whereas MERS-CoV and SARS-CoV-2 have shared similarities in that cases can remain asymptomatic while still spreading the disease. Furthermore, influenza A viruses and SARS-CoV-2 also have a similar characteristic when it comes to transmissibility (127).

In the setting of extensive SARS-CoV-2 transmissions, the possibility of SARS-CoV-2 should be considered in all persons with a fever or lower respiratory infection, because it is challenging to straightforwardly distinguish between seasonal influenza and COVID-19, even if an epidemiologic link cannot be readily established. Furthermore, the timely reporting of cases, updates on clinical status and disposition of patients, the real-time analysis of data, and the appropriate dissemination of information are essential for outbreak-managing decisions.

## Author Contributions

ZA: conceptualization, methodology, investigation, writing—original draft, and visualization. ML: visualization. XW: conceptualization, methodology, project administration, funding acquisition, writing—review and editing, and supervision. All authors contributed to the article and approved the submitted version.

## Conflict of Interest

The authors declare that the research was conducted in the absence of any commercial or financial relationships that could be construed as a potential conflict of interest.

## Supplementary Material

Supplementary Figure 1 The origins and intermediate hosts of SARS-CoV-2, SARS-CoV, and MERS-CoV.

Supplementary Figure 2 Virus-host interaction. Th1, T helper 1; Th17, T helper 17; ACE2, angiotensin-converting enzyme 2; INF-1, interferon 1; INFγ, interferon gamma; DPP4, dipeptidyl peptidase-4; HA, hemagglutinin; NA, neuraminidase; M2e, Matrix 2 protein; MHC-1, major histocompatibility complex class 1.

Supplementary Figure 3 Potential transmission routes of respiratory infection between infected and susceptible individuals (128). Respiratory infections with a droplet nuclei size ≤ 5 μm can travel to a distance ≥1 m. In contrast, respiratory infections with a droplet nuclei size ≥5 μm cannot travel to a distance ≥1 m. Large droplets may fall on different surfaces and infect healthy individuals through direct or indirect contact.

## Abbreviations

SARS-CoV-2: severe acute respiratory syndrome coronavirus 2
SARS-CoV: severe acute respiratory syndrome coronavirus
MERS-CoV: the Middle East respiratory syndrome coronavirus
WHO: world health organization
CDC: center of disease control and prevention
nt: nucleotide
kb: kilobase
KDa: kilodalton molecular weight unit.
