# Supplementary material for: Comparative Review of SARS-CoV-2, SARS-CoV, MERS-CoV, and Influenza A Respiratory Viruses
Source: Front Immunol. 2020 Sep 11;11:552909. doi: 10.3389/fimmu.2020.552909 (PMC7516028; doi:10.3389/fimmu.2020.552909)
Supplement: Supplementary Figure 1 — The origins and intermediate hosts of SARS-CoV-2, SARS-CoV, and MERS-CoV. [file Data_Sheet_1.pdf]

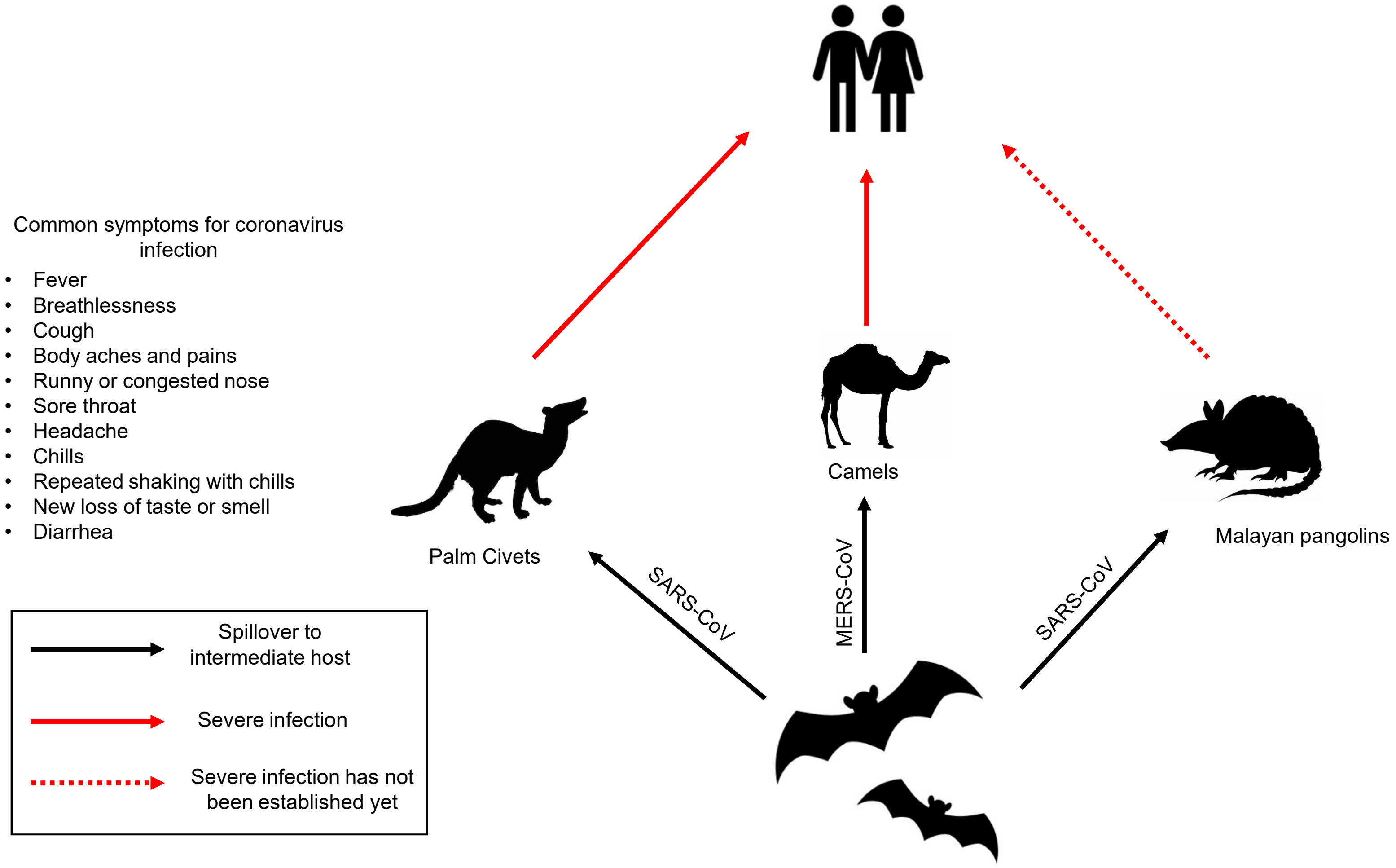

Figure S1: The origins and intermediate hosts of SARS-CoV-2, SARS-CoV, and MERS-CoV

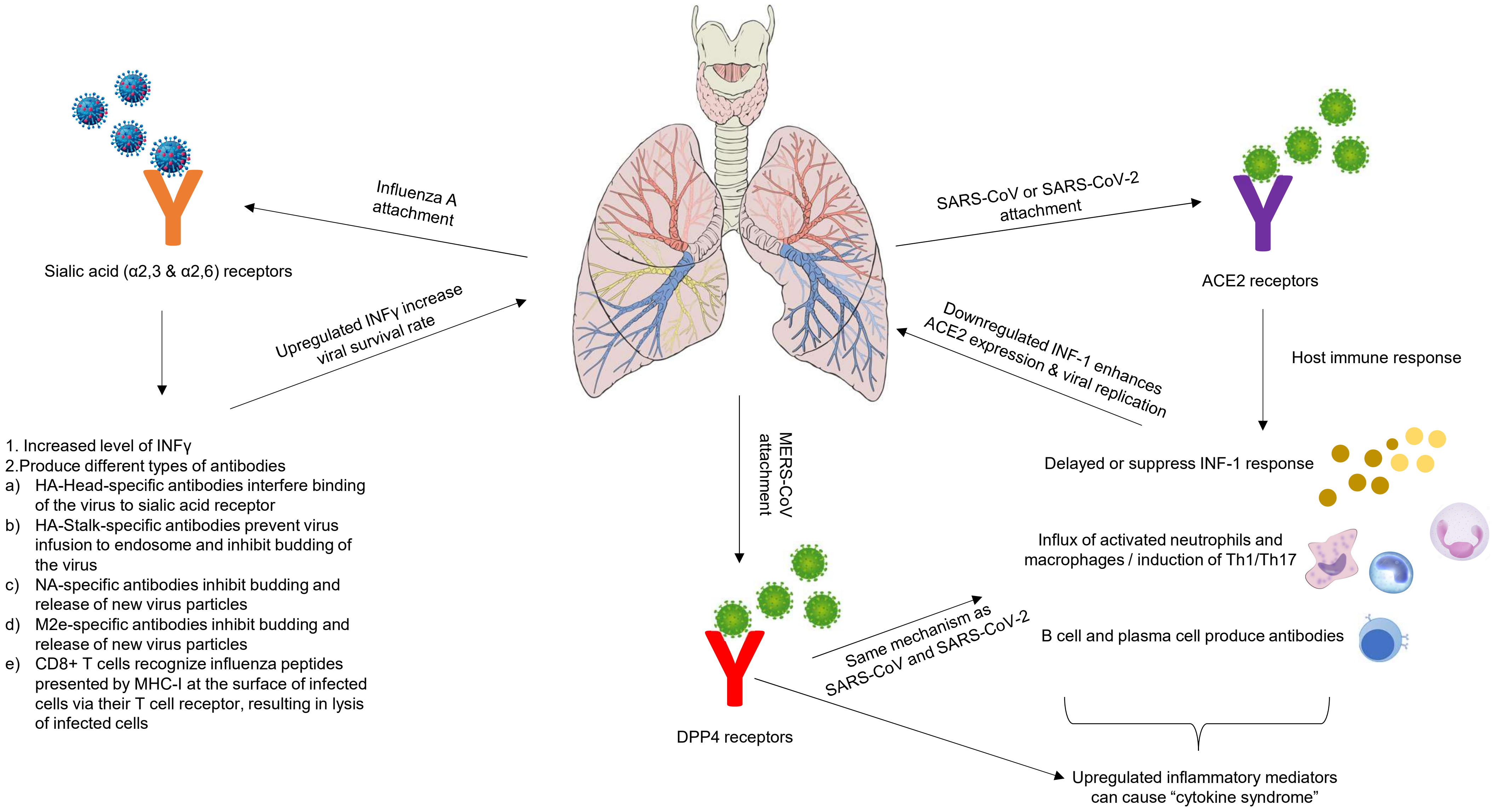

Figure S2. Virus-host interaction. Th1, T helper 1; Th17, T helper 17; ACE2, angiotensin-converting enzyme 2; INF-1, interferon 1; INFγ, interferon gamma; DPP4, dipeptidyl peptidase-4; HA, hemagglutinin; NA, neuraminidase; M2e, Matrix 2 protein; MHC-1, major histocompatibility complex class 1.

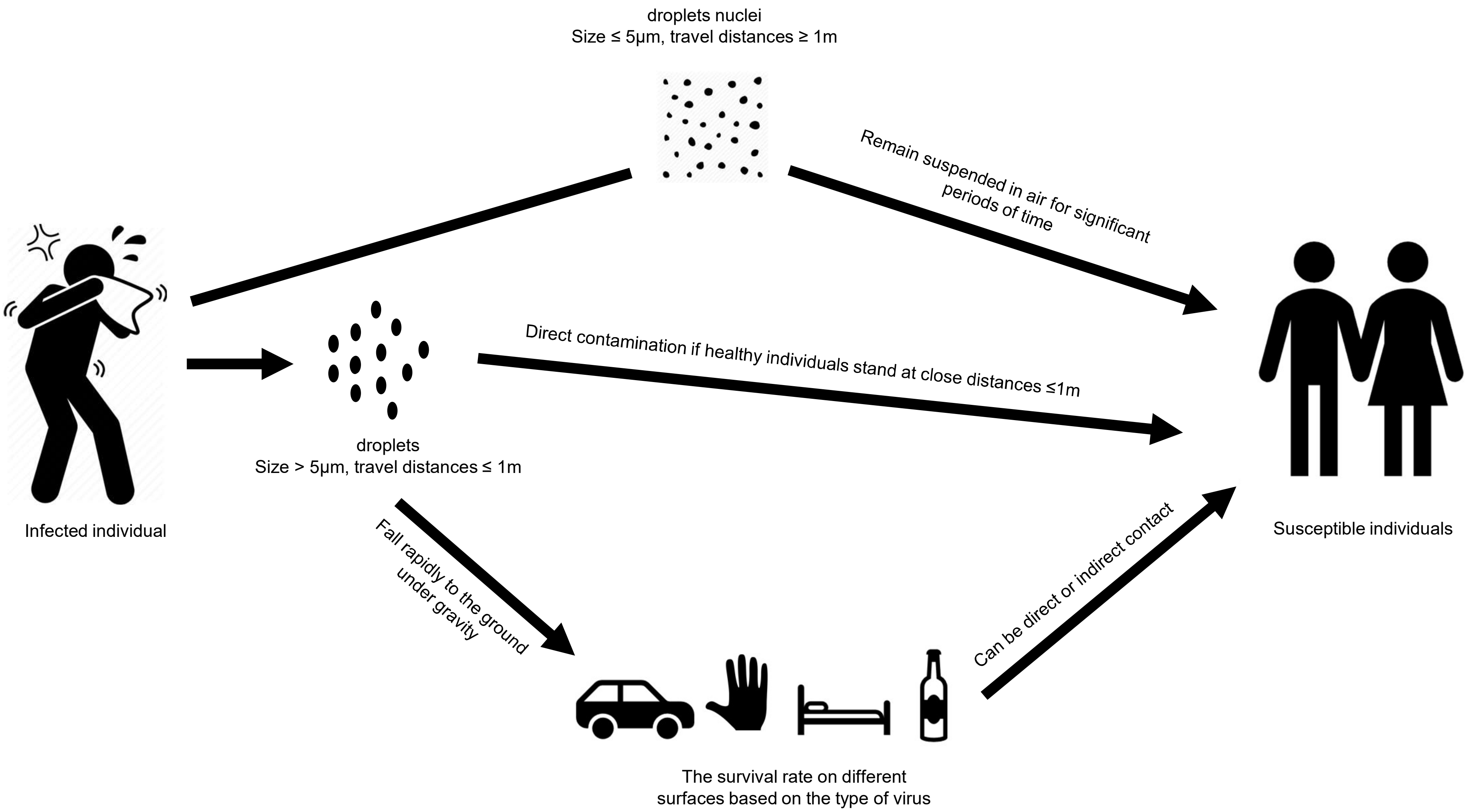

Figure S3: Potential transmission routes of respiratory infection between infected and susceptible individuals (112). Respiratory infections with a droplet nuclei size ≤ 5 µm can travel to a distance ≥ 1 m. In contrast, respiratory infections with a droplet nuclei size ≥ 5 µm cannot travel to a distance ≥ 1 m. Large droplets may fall on different surfaces and infect healthy individuals through direct or indirect contact.
